# Acute Malnutrition in Children: Pathophysiology, Clinical Effects and Treatment

**DOI:** 10.3390/nu12082413

**Published:** 2020-08-12

**Authors:** Valeria Dipasquale, Ugo Cucinotta, Claudio Romano

**Affiliations:** Pediatric Gastroenterology and Cystic Fibrosis Unit, Department of Human Pathology in Adulthood and Childhood “G. Barresi”, University of Messina, 98125 Messina, Italy; dipasquale.valeria@libero.it (V.D.); ugocucinotta@gmail.com (U.C.)

**Keywords:** acute malnutrition, marasmus, kwashiorkor, primary malnutrition, secondary malnutrition, management

## Abstract

Acute malnutrition is a nutritional deficiency resulting from either inadequate energy or protein intake. Children with primary acute malnutrition are common in developing countries as a result of inadequate food supply caused by social, economic, and environmental factors. Secondary acute malnutrition is usually due to an underlying disease causing abnormal nutrient loss, increased energy expenditure, or decreased food intake. Acute malnutrition leads to biochemical changes based on metabolic, hormonal, and glucoregulatory mechanisms. Most children with primary acute malnutrition can be managed at home with nutrition-specific interventions (i.e., counseling of parents, ensuring household food security, etc.). In case of severe acute malnutrition and complications, inpatient treatment is recommended. Secondary acute malnutrition should be managed by treating the underlying cause.

## 1. Introduction

Acute malnutrition is a nutritional deficiency resulting from either inadequate protein or energy intake. In 1959 Jelliffe introduced the term “protein calorie malnutrition”, which has been replaced by “acute malnutrition”. Olsen et al. [1] defined protein energy malnutrition as nutritional deprivation amongst children in developing countries. All terms, though, refer to pediatric undernutrition as a state of nutrition in which deficiency of energy, protein and other nutrients leads to measurable adverse effects on tissue and body functions, and a clinical outcome of growth deviation [2].

According to the American Society of Parenteral and Enteral Nutrition (ASPEN) [3], pediatric malnutrition is defined as “an imbalance between nutrient requirement and intake, resulting in cumulative deficits of energy, protein, or micronutrients that may negatively affect growth, development, and other relevant outcomes.” Based on its etiology, malnutrition is either illness related (one or more diseases or injuries directly result in nutrient imbalance) or caused by environmental/behavioral factors associated with decreased nutrient intake and/or delivery.

Primary acute malnutrition in children is the result of inadequate food supply caused by socioeconomic, political, and environmental factors, and it is most commonly seen in low- and middle-income countries [4,5]. Responsible factors include household food insecurity, poverty, poor nutrition of pregnant women, intrauterine growth restriction, low birth weight, poor breastfeeding and inadequate complementary feeding, frequent infectious illnesses, poor quality of water, hygiene, etc. Therefore, primary acute malnutrition is mostly social rather than biomedical in origin, but it is also multifactorial. For example, poor water quality, sanitation and hygiene practices are increasingly believed to be the cause of the condition called “environmental enteropathy” that contributes to acute malnutrition in childhood [6]. The repetitive exposure to pathogens in the environment causes small intestinal bacterial colonization, with accumulation of inflammatory cells in the small intestinal mucosa, damage of intestinal villi, and, consequently, malabsorption of nutrients, which results in malnutrition.

Secondary acute malnutrition is usually due to abnormal nutrient loss, increased energy expenditure, or decreased food intake, frequently in the context of underlying, mostly chronic, diseases like cystic fibrosis, chronic renal failure, chronic liver diseases, childhood malignancies, congenital heart disease, and neuromuscular diseases [4,5].

Although there may be a lack of consensus on the use of terminology and definition, there is agreement that acute malnutrition should be diagnosed using anthropometrics only (Table 1) [5,7].

The aim of this review is to describe the pathophysiology and main clinical aspects of acute malnutrition in childhood, and to provide an overview of the current recommendations on management based on acute malnutrition type, cause and severity.

### Epidemiology

Acute malnutrition is responsible for almost one third of all deaths in children <5 years of age and causes intellectual or cognitive impairment among those who survive [5]. The estimated number of underweight children (weight-for-age Z score < −2) globally is 101 million or 16%. The prevalence of acute and severe malnutrition among children under 5 is above the World Health Assembly target of reducing and maintaining prevalence at under 5% by 2025.

In studies using various methods of assessing malnutrition, the prevalence of acute malnutrition among hospitalized children in developed countries ranged from 6 to 51% [8,9,10,11,12]. In 2008, Pawellek et al. [11] using Waterlow’s criteria reported 24.1% of pediatric patients in a tertiary hospital in Germany to be malnourished, of which 17.9% were mild, 4.4% moderate, and 1.7% severely malnourished. The prevalence of malnutrition varied depending on underlying medical condition and ranged from 40% in the case of neurologic diseases, to 34.5% for infectious disease, 33.3% for cystic fibrosis, 28.6% for cardiovascular disease, 27.3% for oncology patients, and 23.6% in case of gastrointestinal diseases [11]. Patients with multiple diagnoses were most likely to be malnourished (43.8%). Despite differences in measures of malnutrition, these studies clearly document a significant prevalence of malnutrition even in the developed world [4].

## 2. Pathophysiology

Inadequate energy intake leads to various physiologic adaptations, including growth restriction, loss of fat, muscle, and visceral mass, reduced basal metabolic rate, and reduced total energy expenditure [4,5,6]. The biochemical changes in acute malnutrition involve metabolic, hormonal, and glucoregulatory mechanisms. The main hormones affected are the thyroid hormones, insulin, and the growth hormone (GH). Changes include reduced levels of tri-iodothyroxine (T3), insulin, insulin-like growth factor-1 (IGF-1) and raised levels of GH and cortisol [4]. Glucose levels are often initially low, with depletion of glycogen stores. In the early phase there is rapid gluconeogenesis with resultant loss of skeletal muscle caused by use of amino acids, pyruvate and lactate. Later there is the protein conservation phase, with fat mobilization leading to lipolysis and ketogenesis [13,14,15]. Major electrolyte changes including sodium retention and intracellular potassium depletion can be explained by decreased activity of the glycoside-sensitive energy-dependent sodium pump to increased permeability of cell membranes in kwashiorkor [15].

Organ systems are variably impaired in acute malnutrition [4,15]. Cellular immunity is affected because of atrophy of the thymus, lymph nodes, and tonsils. There are reduced cluster of differentiation (CD) 4 with normal CD8-T lymphocytes, loss of delayed hypersensitivity, impaired phagocytosis, and reduced secretory immunoglobulin A. Consequently, the susceptibility to invasive infections (urinary, gastrointestinal infections, septicemia, etc.) is increased [15,16].

Villous atrophy with resultant loss of disaccharidases, crypt hypoplasia, and altered intestinal permeability results in malabsorption. Other common aspects are bacterial overgrowth and pancreatic atrophy resulting in fat malabsorption; fatty infiltration of the liver is also common [4]. Drug metabolism may be decreased due to decreased plasma albumin and decreased fractions of the glycoprotein responsible for binding drugs [17].

Cardiac myofibrils are thinned with impaired contractility. Cardiac output is reduced proportionate to weight loss. Bradycardia and hypotension are also common in severe cases [4,16]. The combination of bradycardia, impaired cardiac contractility, and electrolyte imbalances predisposes to arrhythmias. Reduced thoracic muscle mass, decreased metabolic rate, and electrolyte imbalances (hypokalemia and hypophosphatemia) may result in decreased minute ventilation and impaired ventilatory response to hypoxia [4,16,18].

Acute malnutrition has been recognized as causing reduction in the numbers of neurons, synapses, dendritic arborizations, and myelinations, all of which resulting in decreased brain size [19]. The cerebral cortex is thinned and brain growth slowed. Delays in global function, motor function, and memory have been associated with malnutrition [19]. The effects on the developing brain may be irreversible after the age of 3–4 years [5].

## 3. Clinical Syndromes

Acute malnutrition pertains to a group of linked disorders that includes kwashiorkor, marasmus, and intermediate states of marasmic kwashiorkor. They are distinguished based on clinical findings, with the primary distinction between kwashiorkor and marasmus being the presence of edema in kwashiorkor [16].

### 3.1. Marasmus

The term “marasmus” is inferred from the Greek word “marasmus”, correlating to wasting or withering. Marasmus is the most frequent syndrome of acute malnutrition [4]. It is due to inadequate energy intake over a period of months to years. It results from the body’s physiologic adaptive response to starvation in response to severe deprivation of energy and all nutrients, and is characterized by wasting of body tissues, particularly muscles and subcutaneous fat, and is usually a result of severe restrictions in energy intake. Children younger than five years are the most commonly involved because of their increased caloric requirements and increased susceptibility to infections [15]. These children appear emaciated, weak and lethargic, and have associated bradycardia, hypotension, and hypothermia. Their skin is xerotic, wrinkled, and loose because of the loss of subcutaneous fat, but is not characterized by any specific dermatosis [4]. Muscle wasting often starts in the axilla and groin (grade I), then thighs and buttocks (grade II), followed by chest and abdomen (grade III), and finally the facial muscles (grade IV), which are metabolically less active. In severe cases, the loss of buccal fat pads gives the children an aged facial aspect. Severely affected children are often apathetic but become irritable and difficult to console [4].

### 3.2. Kwashiorkor

The term “kwashiorkor” derives from the Kwa language of Ghana and its meaning is equivalent to “the sickness of the weaning” [15]. Cicely D. Williams first used the term in 1933. Kwashiorkor is thought to be the result of inadequate protein but reasonably normal caloric intake. It was first reported in children with maize diets (these children have been called “sugar babies”, as their diet is typically low in protein but high in carbohydrate) [4,15]. Kwashiorkor is frequent in developing countries and mainly involves older infants and young children. It mostly occurs in areas of famine or with limited food supply, and particularly in those countries where the diet consists mainly of corn, rice and beans [20]. Kwashiorkor represents a maladaptive response to starvation. Edema is the distinguishing characteristic of kwashiorkor, which does not exist in marasmus [21], and usually results from a combination of low serum albumin, increased cortisol, and inability to activate the antidiuretic hormone. It usually starts as pedal edema (grade I), then facial edema (grade II), paraspinal and chest edema (grade III) up to the association with ascitis (grade IV). Besides edema, clinical features are almost normal weight for age, dermatoses, hypopigmented hair, distended abdomen, and hepatomegaly. Hair is usually dry, sparse, brittle, and depigmented, appearing reddish yellow. Cutaneous manifestations are characteristic and progress over days from dry atrophic skin with confluent areas of hyperkeratosis and hyperpigmentation, which then splits when stretched, resulting in erosions and underlying erythematous skin [4]. Various skin changes in children with kwashiorkor include shiny, varnished-looking skin (64%), dark erythematous pigmented macules (48%), xerotic crazy paving skin (28%), residual hypopigmentation (18%), and hyperpigmentation and erythema (11%) [4].

### 3.3. Marasmic Kwashiorkor

Marasmic kwashiorkor is represented by mixed features of both marasmus and kwashiorkor. Characteristically, children with marasmic kwashiorkor have concurrent gross wasting and edema. They usually have mild cutaneous and hair manifestations and an enlarged palpable fatty liver.

## 4. Assessment

An adequate nutritional assessment includes detailed dietary history, physical examination, anthropometric measurements (including weight, length, and head circumference in younger children) using appropriate reference standards, such as the WHO standard growth charts [22], and basic laboratory indices if possible. In addition, skinfold thickness and mid-upper-arm circumference (MUAC) measurements represent a useful method for evaluating body composition [23].

Questions regarding mealtimes, food intake and difficulties while eating should be part of routine history taking and give a rapid qualitative impression of nutritional intake. For a more quantitative assessment, a detailed dietary history must be taken by recording a food diary or (less commonly) a weighed food intake. This would usually be performed in association with an expert dietician. When considering whether intakes are enough, dietary reference values provide estimates of the range of energy and nutrient requirements in groups of individuals [24].

Accurate measurement and charting of weight and height (length in children < 85 cm, or unable to stand) is essential if malnutrition is to be identified. Clinical examination without plotting anthropometric measurements on growth charts has been shown to be very inaccurate [25]. For premature infants up to two years of age, it is essential to deduct the number of weeks born early from actual (‘chronological’) age in order to obtain the ‘corrected’ age for plotting on growth charts. Head circumference should be routinely measured and plotted in children less than two years old. Head circumference is a reliable index of nutritional status and brain development and is associated with scholastic achievement and intellectual ability in school-aged children [26]. The long-term effects of severe malnutrition at an early age may result in delayed head circumference growth, brain development, and decreased intelligence and scholastic achievement. In a study of 96 right-handed healthy high school graduates (mean ± SD age 18.0 ± 0.9 years) born at term, the interrelationships between head size and intelligence, learning, nutritional status, brain development, and parental head size were examined [27]. In this study, head circumference and brain volume were negatively associated with malnutrition during the first year of life. MUAC has been suggested as a proxy for weight, and head circumference as a proxy for height. MUAC minimally varies during the early years of life. It is simple and accurate, and it predicts malnutrition-related mortality with acceptable specificity and sensitivity [28]. In patients with fluid shifts and edema, MUAC may be a better indicator than weight-for-height for classification of acute malnutrition [28]. Mid-arm muscle circumference (MAMC) may be calculated from MUAC and triceps skinfold using the formula MAMC = MUAC−(triceps skinfold × 0.314). Triceps skinfold alone may be a useful screening tool in children [29]. However, its accuracy in children with extensive muscle wasting may be questionable [30]. The standard criterion for care is to measure recumbent or supine length for infants and children younger than two years of age and standing height for those older than two years. However, it is often difficult to obtain a standing height with acutely ill children, as well as non-ambulatory populations (i.e., cerebral palsy). In such cases, there are various methods available for obtaining linear measurements, such as portable length boards that can convert into stadiometers and therefore could feasibly be used to measure recumbent length for older children (i.e., a measuring table). Notably, if recumbent length and standing height are obtained for the same person, there is a difference of approximately 0.8 cm, with standing height measuring less than recumbent length. Obtaining a recumbent length measurement without proper equipment (i.e., measuring tape on a bed) does not provide accurate results. If a measuring table is not available, it is recommended to obtain an alternative proxy measure of height, such as arm span, knee height, or tibia length. Body mass index (BMI) is calculated as weight in kilograms divided by height in meters squared, and it can be used to express weight adjusted for height. BMI in children is compared with sex- and age-specific reference values.

Laboratory investigations may be helpful in the diagnosis of primary acute malnutrition and are essential in driving treatment in secondary malnutrition [5]. Because nutritional status is an independent predictor of outcome in the ill child, strict attention to indicators of macronutrient (protein) stores and micronutrient (vitamin or mineral) deficiencies is mandatory. Signs and symptoms of specific nutrient deficiencies commonly overlap and multiple deficiencies frequently occur. Assessment of visceral protein stores is commonly made by measuring serum proteins, most commonly albumin, prealbumin (transthyretin) and retinol-binding protein. Interpretation of total protein is predicated on normal globulin levels, limiting its clinical usefulness [5]. Generally, serial measurements of protein status are more meaningful than single values and an understanding of biological half-lives will dictate the frequency of assessment. Their concentrations are susceptible to changes in hydration status and fluid shifts, and these changes may occur rapidly (i.e., increased vascular permeability in case of sepsis or trauma). The decision to evaluate vitamin and mineral stores should consider the underlying pathophysiology (i.e., measurement of fat-soluble vitamins in cases of fat malabsorption, such as celiac disease or cystic fibrosis). An often overlooked group of patients prone to malnutrition are those with absent (surgically resected) or diseased (Crohn’s disease, small-bowel bacterial overgrowth syndrome) terminal ilea. Deficiencies of vitamin B 12, vitamin K and zinc are prevalent in these patients. Finally, some drugs may have potential effects on micronutrient status.

The validity of individual anthropometric measurements may vary based on the population of children. Hence, a combination of measurements obtained by a trained individual in combination with other clinical parameters should guide assessment of nutritional status [5]. Serial anthropometric measurements are mandatory to assess optimal growth during the course of illness.

## 5. Treatment

Management strategies for acute malnutrition depend on the type of malnutrition, identification of its cause, and its severity [5].

### 5.1. Primary Acute Malnutrition

In primary moderate acute malnutrition, management at home is recommended, including counseling of parents, with emphasis on continuing breastfeeding and appropriate complementary feeding (nutrition-specific interventions). Ideally, these children should receive 25 kcal/kg per day of energy in excess of what their healthy peers receive, and their diets should contain animal-source foods rich in essential fatty acids and micronutrients including vitamin A, iron and zinc [5,31,32].

Children with severe acute malnutrition without any complications can be managed in the community with ready-to-use therapeutic food (peanut paste, milk powder, vegetable oil and a mineral and vitamin mix as per WHO recommendations) [33]. Children who have been treated for complications and have appetite can also be treated in the hospital with ready-to-use therapeutic food. Severe acute malnutrition complications (i.e., severe diarrhea, hypoglycemia, hypothermia, pneumonia, urinary tract infection, sepsis, etc.) require hospitalization until children are ready to continue management at home [5].

The stabilization phase of treatment for complications includes [32]: (i) treat hypoglycemia with oral or intravenous glucose if the child is lethargic, unconscious or convulsing; (ii) treat and prevent hypothermia; (iii) treat shock; (iv) treat and prevent dehydration (preferably with the special rehydration solution for malnutrition, ReSoMal, 5 mL/kg body weight orally or by nasogastric tube every 30 min for the first 2 h, then at 5–10 mL/kg/h in alternate hours for up to 10 h); (v) treat and prevent infection. Oral amoxicillin at 15 mg/kg eight-hourly for five days is suggested if the child has no complications, while ampicillin at 50 mg/kg i.m./i.v. six-hourly for two days, followed by oral amoxicillin for five days and gentamicin at 7.5 mg/kg i.m./i.v. once daily for seven days in case of complication. If the child fails to improve clinically by 48 h or deteriorates after 24 h, a third-generation cephalosporin (i.e., ceftriaxone at 50–75 mg/kg i.v. or i.m. once daily) may be started with gentamicin; (vi) start careful feeding. Feeding should be started as soon as possible after admission with the WHO-recommended milk-based starter formula F-75, which contains 75 kcal/100 mL and 0.9 g protein/100 mL. The feeding frequency is gradually decreased (Table 2). 

The refeeding syndrome is due to the sudden availability of glucose, leading to inhibition of gluconeogenesis and an insulin surge. This causes rapid intracellular influx of potassium, magnesium, and phosphate and therefore low serum levels and poor myocardial contractility [4,34]. This clinical syndrome, which can manifest with excessive sweatiness, muscle weakness, tachycardia, and heart failure, may be prevented by avoiding rapid carbohydrate feeding, supplementing phosphate and thiamine during the initial increase in nutritional intake, and monitoring the patient carefully for alterations in serum phosphate, potassium, and magnesium; (vii) achieve catch-up growth, which starts when the energy intake is >150 kcal/kg/day. In settings where a program for the community-based management of severe acute malnutrition with ready-to-use therapeutic food is not available, F-100 is used. Feeding is gradually increased to achieve a rapid weight gain of >10 g/kg/day. The WHO recommends the milk-based diet for nutritional rehabilitation F-100, which contains 100 kcal and 2.9 g protein/100 mL.

Chronic malnutrition needs nutrition-sensitive interventions scaled up at the national or regional level, including ensuring household food security, safe water, proper hygiene, female education, creating proper livelihoods, social protection schemes, etc. [5,35]. Growth monitoring should be implemented at the community level, where the nutritional status of infants and young children should be assessed every one–three months and their growth empowered through counseling of parents, even before malnutrition occurs.

### 5.2. Secondary Acute Malnutrition

For the management of secondary acute malnutrition, it is crucial to identify the underlying disease by history taking, examination and laboratory investigations [5]. Exclusive breastfeeding for the first six months along with iron supplementation is adequate for preterm and low-birth-weight infants. They are at risk of necrotizing enterocolitis if aggressive enteral feeding is delivered. In mild inflammatory bowel disease or disease in remission, the intake of a normal diet can be suggested. Commercial, specially prepared liquid formulas are helpful for some patients with inflammatory bowel disease [36]. In advanced chronic liver disease, the diet may need to be protein sparing for the prevention of hyperammonemia. A combination of lipids and carbohydrates with a minimal amount of protein should be used. Another important feature in chronic liver disease is decreased bile salt excretion into the small intestine, which can cause malabsorption of fats and fat-soluble vitamins. This can be faced by using medium-chain triglycerides as the source of dietary fat, since they do not depend upon bile salts for absorption. Water-soluble forms of the usually fat-soluble vitamins (A, D, E and K) should be used. Children with chronic renal disease may benefit from high-energy as well as high-quality protein in quantities that will not induce or worsen uremia [5]. Children with congenital heart disease need to be provided with sufficient energy and protein without increasing the fluid volume too much. They have reduced food intake due to fatigue, dyspnea and frequent lung infections. The heart failure and increased breathing efforts induce a hypermetabolic state that further increases the demand for more nutrients [5]. Children with cancer, chemotherapy, radiation, surgery and infections often present with cachexia, due to tumor necrosis factor-α and tumor metabolites. The diet has to be modified to cater to the increased caloric needs. Parenteral nutrition can be used to improve nutrition in case of poor tolerance to large volumes of enteral feeds.

The principles of management of severe malnutrition resulting from the underlying diseases mentioned above are similar to those for primary severe acute malnutrition.

### 5.3. Acute Malnutrition Management in Humanitarian Crises

In humanitarian crises, supplementary feeding is considered the main strategy for preventing and treating moderate acute malnutrition. According to the Sphere guidelines [37], two types of supplementary feeding programs can be implemented: (i) blanket supplementary feeding programs for preventing, and (ii) targeted supplementary feeding programs for treating moderate acute malnutrition and preventing severe acute malnutrition. The use of each depends on the severity of acute malnutrition, vulnerable population groups and the risk of an increase in acute malnutrition.

Blanket supplementary feeding programs are recommended where food safety is low and there is a need to expand interventions beyond only moderate acute malnutrition cases. They should be accompanied by general food distribution targeting affected households. Defined impact indicators for blanket supplementary feeding programs do not exist, but it is important to check coverage, adherence, acceptability and rations provided.

The indicators for managing moderate acute malnutrition mainly refer to targeted supplementary feeding. The main aim of a targeted supplementary feeding program is to prevent the moderately malnourished becoming severely malnourished and to rehabilitate them. These types of program usually provide a food supplement to the general ration for moderately malnourished individuals, for pregnant and nursing mothers, and other at-risk individuals.In conclusion, acute malnutrition is a nutritional deficiency resulting from either inadequate energy or protein intake, with variable clinical presentation. Clinical examination and careful measurement of growth status and reference to standard growth charts is essential in order to identify children with acute malnutrition. Most children with primary acute malnutrition can be managed at home, while those with severe acute malnutrition and complications require treatment in a hospital; those without complications can be treated at home with ready-to-use therapeutic food. The management of secondary malnutrition is mainly based on treating the underlying cause (malabsorption, infections, etc.).

## Figures and Tables

**Table 1:** New terms used for childhood malnutrition (adapted from Koletzko, B. et al. (eds), 2015) [5].

Term	Definition
Moderate acute malnutrition	Mid-upper-arm circumference greater or equal to 115 mm and less than 125 mm Weight-for-height Z score < −2 but > −3
Severe acute malnutrition	Mid-upper-arm circumference < 115 mm Weight-for-height Z score < −3 Bilateral pitting edema Marasmic kwashiorkor
Global acute malnutrition	The sum of the prevalence of severe acute malnutrition plus moderate acute malnutrition at a population level

**Table 2:** Feeding of children with severe acute malnutrition (adapted from Koletzko et al. (eds.), 2015) [5].

Days	Frequency	Volume/kg Per Feed, mL	Volume/kg Per Day, mL
1–2	2-hourly	11	130
3–5	3-hourly	16	130
6–7	4-hourly	22	130
